# Supplementary material for: Effect of the oil from the fatty tissues of Crocodylus siamensis on gut microbiome diversity and metabolism in mice
Source: PLoS One. 2023 Jul 28;18(7):e0289073. doi: 10.1371/journal.pone.0289073 (PMC10381048; doi:10.1371/journal.pone.0289073)
Supplement: Attachment — Submitted filename: Response to Reviewers.docx [file pone.0289073.s004.docx]

**Response to Reviewers**

Dear Editor and Reviewers,

We would like to thank you so much for your organization and the reviewers’ comments concerning our manuscript entitled “Effect of the oil from the fatty tissues of *Crocodylus siamensis* on gut microbiome diversity and metabolism in mice” (PONE-D-23-11866R1). All comments are valuable and very helpful for revising and improving our manuscript. We have made corrections carefully and marked the revised portion with red color in the paper. We hope that our revised manuscript will improve to be satisfactory and meet with approval.

Response to reviewers

**Reviewer #1:**

Reviewer #1: For comment #1: The authors have corrected the conclusion in the the discussion but not in the abstract.The authors should modify the abstract to be consistent. Specifically, the conclusion "Thus, CO may be an alternative fat source to improve the host metabolism by maintaining the gut flora" is not supported as improvement of the host metabolism and is not demonstrated.

**Response:** Thank you for your concern and nice recommendation. We have checked, downplayed, and revised the conclusion in the abstract already as follows: “Thus, CO may be an alternative fat source for preserving host metabolism and gut flora.”, please see pages 3, lines 51-52.

**Reviewer #3:**

The authors have clearly done their best to take into account the referees' comments when revising the manuscipt. As a result, the scientific quality of the manuscript has considerably improved. This particulalrly holds true for the conclusions of the experimental findings, which have been presented in a less exaggerated, more realist fashion.

**Response:** Thank you for your concern and nice recommendation. The scientific quality and writing in our results, discussion, and conclusion have been improved in a more realist fashion.

A few examples in only the Abstract are:

- line 28: 'fat tissues'; must be changed to 'fatty tissues'

- lines 29-30: 'has been reported to improve inflammation, toxification, and energy metabolism' must be changed to, for instance, 'has been reported to reduce inflammation, counter toxification, and improve energy metabolism'

- line 31: 'on gut microbiota (GM) with metabolic changes in mice' must be changed to, for instance, on gut microbiota (GM) in labotratory mice as well as the accompanying metabolic changes in the animals'

- lines 33-34: 'administrated with sterile water (control [C]), 1 and 3% (v/w) CO (CO-low [CO-L] and CO-high [CO-H]), or 1 and 3% (v/w) palm oil (PO-low and PO-high) for 11 weeks' must be changed to 'administrated either sterile water (control [C]); 1 or 3% (v/w) CO (CO-low [CO-L] and CO-high [CO-H]), respectively); or 1 or 3% (v/w) palm oil (PO-low and PO-high, respectyively) for 11 weeks'

- line 36: 'Colon samples' must be changed to 'Samples from colon tissue'

- line 37: clarify GM analyses

- lines 38-40: 'CO-L and CO-H groups showed a significant reduction in food intake, but they had no effect on calorie intake when compared to the C group' must be changed to, for instance, Food intake by the mice in the CO-L and CO-H groups was statistically significantly less when compared to that by the the animals in the C group. However, neither CO treatment had a statistically significant effect on calorie intake when compared to the controls'.

**Response:** Thank you for your concern and nice recommendation. We have checked and revised already, please see the revised Abstract (pages 2-3; lines 26-52).
